# Coordinated multidisciplinary care for ambulatory Huntington's disease patients. Evaluation of 18 months of implementation

**DOI:** 10.1186/1750-1172-6-77

**Published:** 2011-11-18

**Authors:** Ruth B Veenhuizen, Branda Kootstra, Wilma Vink, Janneke Posthumus, Pleuntje van Bekkum, Margriet Zijlstra, Jelleke Dokter

**Affiliations:** 1 Zorggroep Noorderbreedte, Oostergostraat 52, 9001 CM Grou, The Netherlands; 2 Representative of the Dutch Huntington Association, Postbus 304702500 GL Den Haag, The Netherlands

**Keywords:** Huntington's disease, Coordinated care, Multidisciplinary care, Outpatient

## Abstract

**Background:**

A multidisciplinary outpatient department was set up in the northern part of the Netherlands because of a local lack of adequate treatment and care for Huntington's disease (HD)patients. Outreaching multidisciplinary care is a novel way to optimise functioning and quality of life of HD patients. The vast majority of patients want to stay home as long as possible. Huntington's disease is a devastating neurodegenerative disorder leading to complete disability and long term residence in a specialised institution. In this paper we outline this new type of treatment and give the results of 1.5 year, we also present the results of an inquiry on the appreciation of the working method.

**Methods:**

In the first project half (1.5 yr) 28 patients were seen as had been anticipated. The multidisciplinary team consisting of an institutional physician, a psychologist, a speech and language therapist, a social worker, an occupational therapist and a case manager, assesses the stage of the disease and formulates, coordinates and implements the individual care and treatment plan in the home situation. After 1.5 year a questionnaire on the appreciation of the department was sent to patients, caregivers, healthcare professionals, the lay organisation and Dutch "experts in the field".

**Results:**

For the 28 HD patients a total of 242 problems and actions were verbalised in the care plan, which was accepted by the majority of the patients. Especially informal caregivers, the lay organisation and the Dutch "experts in the field" were enthusiastic on the outreaching and multidisciplinary nature of the department. The verdict over the continuance of the clinic was positive and unanimous.

**Conclusions:**

We concluded that coordinating outreaching multidisciplinary care from an outpatient clinic into the dwelling place of the patient is feasible and appreciated.

## Background

Huntington's disease (HD) is a dreadful disorder with a slow midlife onset and a continuously progressive neurodegenerative nature [1]. It diminishes motor, cognitive, behavioural and social functions of the patient and finally leads to complete dependence of care and death. Because of the mixture of symptoms, its progressive course and the autosomal dominant heredity, the disease has great impact upon spouses and other close relatives and friends. Until now there is no cure for HD, and the available medication to attenuate symptomatology is often accompanied by side-effects. This depressing situation and a lack of adequate care and knowledge in the northern part of the Netherlands has led to the development of a multidisciplinary team working on an out-patient basis with the focus on functional optimisation and quality of life of the HD patient and his/her close relatives [2]. The idea of this project is to get to know the person behind the disease and enable this person to be seen and to live with HD. Staying home as long as possible is one of the major goals of HD-patients and the multidisciplinary team seeks to support patients and their caregivers in this aim [3]. The project (outpatient clinic) was financed through an innovation programme initiated by The Dutch Healthcare Authority (NZa), this board supervises all Dutch healthcare providers and insurers. The therapies and care at home were regularly financed from the Dutch General Exceptional Medical Expenses Act (AWBZ). In this paper the method of the clinic is outlined and results of 18 months of the project are described. After 18 months "experts-in-the-field", patients, caregivers and health care professionals were questioned on the working procedures of the clinic. The results of this survey are given below.

## Methods

### Setting and Protocol

In the northern part of the Netherlands each year about 20 patients are diagnosed with HD. These patients can be referred to the multidisciplinary HD clinic. Patients can also find the clinic on internet, or can be referred by their general practitioner(GP) or their neurologist. The patient accompanied by a close relative or caregiver visits the clinic for a full morning of assessment. The clinic team consists of an elderly care (institutional) physician, a psychologist, an occupational therapist, a speech and language therapist, a social worker and a case manager (specialised nurse). Prior to the visit to the clinic the psychologist always visits the patient at home with the objective to lower the threshold of the clinic and to observe the patient and family members in the home situation. During the clinic visit, disease burden and functional consequences of HD are assessed by each member of the team for patient and caregiver separately. The occupational therapist and the speech and language therapist have lunch with patient and caregiver for observation of manual dexterity and swallowing. The occupational therapist visits the patient at home afterwards to investigate safety in and around the house. Functional skills concerning activities of daily living and mobility are also examined. The social worker offers the making of a life book to the patient and caregiver. The aim of this life book is to get to know the person behind the disease, to build a fruitful relation with the patient and to ease communication in the later stages of the disease. The different tasks of the members of the multidisciplinary team are shown in Table 1.

**Table 1:** Different tasks of team members during intake and control visit.

Team member	Tasks
Elderly care physician	Assessment of the disease and the consequences for functional capacity (somatic, activities of daily living, social, psychological, communication) Mini Mental State Exam Neurological screening Measurement of weight Composition of the care plan and coordinating implementation Medication prescription, crisis intervention Information, support

Psychologist	Visit at home with information on HD and the HD clinic Psychological assessment on behavioural and communicative problems in the home situation Cognitive assessment Mood assessment Experience investigation Personal strong and weak points in relation to spouse, children, relatives Ego-support, therapy Supervision for patient and relatives

Speech and language therapist	Swallow investigation Speech and language processing assessment Information, advice, therapy for swallowing, speech and language

Occupational therapist	Assessment of manual dexterity and activities of daily living Home visit with safety investigation Observation of activity (showering, cooking, biking) Advice and help or training with adjustments and aids Therapy in organising and planning and executing activities

Social worker	Life book Assessment of carrying capacity of patient and caregiver(s) Social support, advice and help in organising social network Advice on financial problems Ego-support

Case manager	Responsible for organisation of care plan in home situation First contact person for patient and caregivers Linking pin to the multidisciplinary team members Information and education to district nursing teams Advice on regulations concerning care etc.

At the end of the morning the multidisciplinary team deliberates and composes a care plan with specialised implementation at home. The elderly care physician is responsible for the contents of the plan and the case manager is responsible for the organisation of the implementation. A care plan consists of the identified problems and their goals which should be strived for, in the coming half year. Each goal is connected to one or more persons (formal professionals and informal) who are going to execute the plan. *For instance the problem is "fall in the shower", spouse worries about safety and patient wants to shower on his own. The following goal is then verbalised: "to shower safely as long as possible on one's own" and the occupational therapist will execute this plan and will train the patient to do this in a structured and safe way*. This is preferably an occupational therapist living nearby. This therapist is instructed and guided by the occupational therapist from the clinic. The complete plan is provided to the patient and is executed on patient agreement; it also includes explanation of symptom management to patient and caregivers, both family and professional. In the vast majority of cases there is extensive coordination of care at home by the case manager. The primary goal of the plan is optimal functionality combined with a good quality of life for patient and family. Patient, family, GP, district nurses and local therapists are supported in the execution of the care plan which is divided in 4 categories; physical, housing/living, social and mental conditions. Health care professionals were invited to communicate upon the intended goals and if desired they were supported in obtaining the therapy objectives. If necessary, the clinic team could easily be augmented by trained professionals from the nursing home (e.g. physical therapist, dietician, pastor). Each half year the patient is reassessed and the care plan is evaluated and adjusted to the actual situation [2].

### Method, data collection and analysis

After 18 months the project was evaluated through an inquiry into the appreciation of this new type of outreaching care for HD patients. For this survey a total of 124 questionnaires with a mixture of 10 open and multiple choice questions was sent to 24 patients, 20 direct caregivers, 60 involved healthcare professionals and 20 nursing teams. Five Dutch "Experts-in-the-field" and the lay organisation were asked to give their opinion on the working method of the outpatient department. Assessment of stage of disease was scored according to Shoulson and Fahn [4]. Data are presented as mean ± SD, when interesting a range is also given. Results of the questionnaire are summarised in percentages of responders.

## Results and Discussion

### Patients and method

Twenty eight patients were seen in 18 months. The patients were living in four provincies of the northern part of the Netherlands. Roughly 1/8 of the Dutch population (total 16 million) lives in this rural area which covers 1/4 of the surface area of the country. Of the 28 patients 8 visited a specialised neurologist besides our clinic. Table 2 shows the baseline characteristics of the patients. The total number of problems set at first visit to the clinic is shown in Table 3. Of 28 patients 21 agreed on the execution of all the interventions of the care plan, 7 (25%) of them were reluctant to accept the full plan, they agreed on parts of the plan. Among the refused interventions was; starting with therapy, starting day care and testing driving proficiency. The implementation of the interventions led to many contacts with healthcare professionals, probation officers, municipal officials, regulation officers etc. Each half year the disease burden was assessed and the care plan was evaluated with the patient and caregivers, informal and professional. Two patients of the 28 withdrew from the controls. Three patients were institutionalised, of these patients one died, 82 years old, after 3 months of admission as a consequence of aspiration pneumonia.

**Table 2:** Baseline characteristics of the patients.

Characteristic	n
Male	10 (mean age 63.3 ± 8.7 yrs, range 52-82)
Female	18 (mean age 53.9 ± 13.5 yrs, range 24-73)
Living with partner	17
Living alone	9
Living in an institution	1
Shoulson stage [4]	
1	0
2	4
3	5
4	18
5	1

**Table 3:** Frequency of problems (with examples in italics) formulated for 28 HD patients arranged per category.

Category	Frequency
**Physical**	88
*swallowing, falling, fatigue, weight loss, hygiene*,	
*clothing, eating, drinking*	
**Housing/living conditions**	43
*cooking, stairs, toilet, bathroom, garden*	
**Social**	68
*relation to caregiver, meaningful daily activity*,	
*going out in society*	
**Mental**	43
*Cognition, depression, aggression, diminished impulse control, apathy*	

Total	242
Per patient	8.6 (range 4-11)

### Evaluation of the project

Of a total of 130 questionnaires 77 (59%) returned a reply. Table 4 summarises the multiple choice questions and their answers. Twenty four patients were inquired, 4 patients were not eligible to fill out a form. Of these 24 patients 15 (63%) responded. The informal caregivers (n = 20) were involved with care on a daily basis, of them 16 (80%) returned the questionnaire. Of 20 nursing teams 8 (40%) responded and of 60 health care professionals 32 (53%). The 5 "experts in the field" whom we asked, all returned a reply. The lay organisation also outlined their evaluation of the working method of the clinic. All responders (77) were unanimous that the clinic should continue its service, they even suggested to increase the service with more mutual deliberation and more locations. The last question in the inquiry was to grade the treatment by the clinic from 0 (worthless) till 10 (excellent) see Table 5.

**Table 4:** Questions and answers arranged to groups of respondents.

Questions	Answers*
**Patients**	
1.I derive benefit from the treatment from the HD clinic	81% agree 6% don't agree 13% don't know
2. The professionals from the clinic are well informed about HD	100% agree
3. My health care professionals at home collaborate well with the professionals from the HD clinic	80% agree 0% don't agree 20% don't know
4. Through the meddling of the HD clinic my quality of life is	53% increased 27% even 13% don't know 0% decreased

**Informal caregivers**	
1. I have confidence in the treatment given by the HD clinic	93% agree 7% don't agree
2. The professionals from the HD clinic are well informed about HD	93% agree
3. The health care professionals at home collaborate well with the professionals from the HD clinic	79% agree 0% don't agree 14% don't know
4. I am adequately supported by educated health care professionals	93% agree 7% don't know
5. Partly due to the HD clinic my partner is still at home	79% agree 7% don't agree 7% don't know

**Nurses and day care workers**	
1.The professionals from the HD clinic support me adequately to implement the care plan coordinated by the clinic	100% agree
2. The professionals from the HD clinic are well informed about HD	100% agree
3. The method of working suits well with the regular care I give to my patient	100% agree
4. My collaboration with the professionals from the HD clinic is	71% amply sufficient 29% sufficient
5. My knowledge on HD has increased due to the HD clinic	100% agree

**Healthcare professionals**	
1. The contribution of the HD clinic to the care for HD patients is evident to me	70% agree 10% don't agree 20% don't know
2. The method of working suits well with the regular care I give to my patient	77% agree 3% don't agree 20% don't know
3. I think it is good that HD treatment of our mutual HD patient is coordinated through the HD clinic	80% agree 3% don't agree 17% don't know
4. Collaboration with the professionals from the HD clinic is	40% amply sufficient 53% sufficient 3% insufficient
5. My knowledge on HD has increased due to the HD clinic	40% agree 37% don't agree 20% don't know

* When the added numbers do not equal 100% one or more respondents did not fill out the question.

**Table 5:** Grades (mean plus standard deviation, SD) given by the inquired groups.

Groups	Grade ± SD; 0 = worthless, 10 = excellent
Patients	7.9 ± 1.7

Caregivers	8.5 ± 1.2

Healthcare professionals (nurses incl.)	7.9 ± 1.0

Total	8.0 ± 1.2

One or two open questions were added to the questionnaire for patients and caregivers on specific experience with HD and on the need for care. Patients and caregivers gave quotes on these questions.

What do you experience as worst on having HD? Quotes from patients: *"little contact", "chorea", "loss of weight", "fatigue", "anger outbursts", "physical and mental deterioration", "losing my memory", "that you don't know how bad it will be", "the loss of freedom when you're not allowed to drive a car anymore", "that I have it"*.

What is most essential in the care for an HD patient? Quotes from patients: "*To be treated like a normal person", "Being understood", "Staying home as long as possible", "Personal support at home" , "knowledge of HD", "good treatment"*. Most important quotes from informal caregivers: *" Accompaniment by professionals who know what the disease means", "understanding", "warmth", "support", "treating the HD patient as a normal person"*.

"Experts in the field" and the lay organisation were asked for their opinion on good care for the ambulant HD patient. The continuous thread through their answers is the emphasis on the multidisciplinary approach because of the complex nature of the disease. Psychiatric, neurological, psychological, paramedical and nursing treatment and care should be integrated. The "experts" also stipulate the importance of research and education.

## Discussion

Many authors have stood up and set criteria for multidisciplinary treatment of HD [1,5-7]. These criteria have formed our coordinated multidisciplinary approach. In this paper our service is elucidated. Results of 18 months multidisciplinary care and of an inquiry on the appreciation of the approach by patients and caregivers are given. From the results of our service we conclude that organising this type of care is feasible. Although 18 months is a relatively short period of time for HD, the results underline the suitability of the multidisciplinary approach for HD. Nance has put three goals into words in the definition of care for HD: reduction of burden of symptoms, maximising function and minimising crises [7]. When these goals are obtained a contribution is made to quality of life of patients and their caregivers. Again for evaluation of quality of life the time span of 18 months (and less) is short, yet the results of the survey seem positive in terms of quality of life. Research on efficacy (e.g. reduction in crisis admissions) in obtaining the abovementioned goals is necessary. Until now there is no literature available on the exact contents of multidisciplinary treatment and on effectiveness of this type of treatment.

The majority of our patients live at home and suffer with their families from serious symptoms of HD (Shoulson stage 4) [4]. Until the visit to our outpatient department less than 30% of them were seeing a trained neurologist. Working in a chain of therapists and caregivers, professional and informal, on implementing the care plan is often successful, although sometimes difficult to organise. In a larger area without a specialised nursing home, care for patients with HD can only be offered in the home situation. Trained doctors and therapists are necessary to identify problems and their solutions, because patients and caregivers do not easily complain probably as a consequence of anosognosia [3,8], family history and shortage of knowledge on solutions and efficacy of therapy. It appears that families often don't know where to go with their problems. In our outpatient clinic we offer patients, families and professionals 1 person, the case manager, to refer to. She functions as a linking pin and she can always discuss a problem in the multidisciplinary deliberation. As we train spouses how to cope with the HD-patient and how to understand the difficulties of behaviour, we think that crises can be foreseen and sometimes be prevented.

In this study 59% reacted on our inquiry, which is of course only a little majority. As the survey was anonymous we could not send a reminder to the non-responders and we have no clues for the reasons of non responding. For the 5 "experts in the field" and the lay organisation the reply percentage was 100% and for the informal caregivers (mostly spouses or very close friends) the response was 80%. And because of these response rates, we consider the survey for these groups as representative. The overall tendency in the answers of the healthcare professionals supports the results of the representative groups.

The results of our inquiry show the appreciation especially by caregivers on our offered care plan. Patients and caregivers, lay and professional, even request more interaction with the outpatient clinic. Multidisciplinary deliberation and agreement on the composition of the care plan is of major importance for effective treatment and accompaniment of HD patients and their families. We believe this is an important but difficult success factor of our clinic. It is therefore that we strive for a network of collaborating trained therapists treating the patients in their dwelling place.

## Conclusion

Outreaching coordinated multidisciplinary care for ambulatory HD patients is realisable and appreciated by patients, caregivers and health care professionals. The majority of caregivers hold the opinion that the patient stays at home longer due to the coordinated multidisciplinary care. Involved "experts in the field", patients, caregivers, health care professionals and the lay organisation hold the opinion that the HD outpatient clinic should continue its service. Although the number of patients is small we think the results are important for the evaluation of the project and for future research on efficacy of multidisciplinary treatment of HD. Minimising crises and maximising function should lead to postponement of residential care and reduction of crisis admissions and thereby to a decrease in costs and an increase in quality of life. These end-points could be the focus of future research.

## List of abbreviations

HD: Huntington's disease; GP: General practitioner.

## Competing interests

The authors declare that they have no competing interests.

## Authors' contributions

RV is head of the outpatient department and acquired, analysed and interpreted the data, RV also wrote the manuscript. BK, WV, JP, PB, MZ and JD are all members of the clinic team and acquired the data and read and approved the final manuscript.
